# Supplementary material for: Asynchronous magnetic resonance elastography: Shear wave speed reconstruction using noise correlation of incoherent waves
Source: Magn Reson Med. 2022 Oct 27;89(3):990–1001. doi: 10.1002/mrm.29502 (PMC9792433; doi:10.1002/mrm.29502)
Supplement: Supplementary file 1 — DATA S1 MATLAB code used to generate the numerical simulations of Figure 1 [file MRM-89-990-s002.zip › k-Wave/helpfiles/example_tvsp_3D_simulation.html]

Simulations In Three Dimensions Example (k-Wave)


# Simulations In Three Dimensions Example

This example provides a simple demonstration of using k-Wave for the simulation and detection of a time varying pressure source within a three-dimensional heterogeneous propagation medium. It builds on the Monopole Point Source In A Homogeneous Propagation Medium Example and Simulations In Three Dimensions examples.

- Open the file in the MATLAB Editor
- Run the file in MATLAB

## Contents

- Defining the time varying pressure source
- Running the simulation

## Defining the time varying pressure source

As in one and two dimensions, a time varying pressure source is defined by assigning a binary matrix (i.e., a matrix of 1's and 0's with the same dimensions as the computational grid) to `source.p_mask`, where the 1's represent the grid points that form part of the source. The time varying input signal is then assigned to `source.p`. This can have any number of time points (it doesn't need to match `kgrid.Nt`) and can be defined as either a single time series (in which case it is applied to all of the source points), or a matrix of time series following the source points using MATLAB's standard column-wise linear matrix index ordering. Here a sinusoidal input is assigned to a square source element. To remove frequencies not supported by the spatial grid, the input should first be filtered using `filterTimeSeries` (see the Filtering A Delta Function Input Signal Example for more information).

```
% define a square source element
source_radius = 5;  % [grid points]
source.p_mask = zeros(Nx, Ny, Nz);
source.p_mask(Nx/4, Ny/2 - source_radius:Ny/2 + source_radius, Nz/2 - source_radius:Nz/2 + source_radius) = 1;

% define a time varying sinusoidal source
source_freq = 2e6;  % [Hz]
source_mag = 1;     % [Pa]
source.p = source_mag * sin(2 * pi * source_freq * kgrid.t_array);

% smooth the source
source.p = filterTimeSeries(kgrid, medium, source.p);
```

A plot of the source and sensor masks using `voxelPlot` is shown below.


## Running the simulation

To allow visualisation of the source elements within the grid, the source mask is assigned to the optional input parameter `'DisplayMask'`. This mask is overlaid onto the plot during the simulation. The optional input `'DataCast'` is also set to `'single'` to reduce the computation time.

```
% input arguments
input_args = {'DisplayMask', source.p_mask, 'DataCast', 'single'};

% run the simulation
sensor_data = kspaceFirstOrder3D(kgrid, medium, source, sensor, input_args{:});
```

A plot of the display during simulation is shown below.

An animated slice-by-slice visualisation of the final pressure field can be viewed using `flyThrough`.

```
% view final pressure field slice by slice
flyThrough(p_final);
```
